# Supplementary material for: Influence of the Levels of Arsenic, Cadmium, Mercury and Lead on Overall Survival in Lung Cancer
Source: Biomolecules. 2021 Aug 5;11(8):1160. doi: 10.3390/biom11081160 (PMC8392714; doi:10.3390/biom11081160)
Supplement: Supplementary file 1 [file biomolecules-11-01160-s001.zip › biomolecules-1274222-supplementary.pdf]

Supplementary Materials

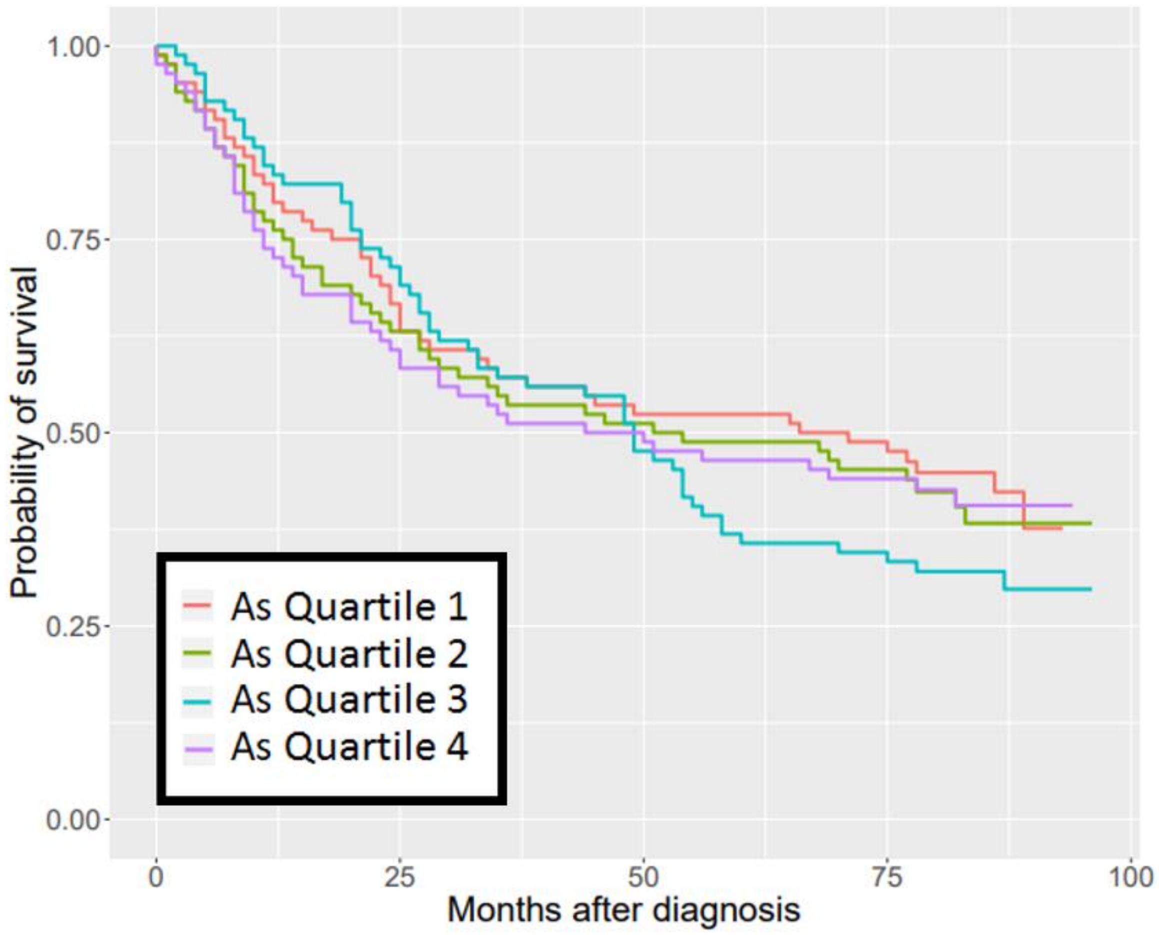

Figure S1. 96-months overall survival by arsenic in the group of all lung cancer.

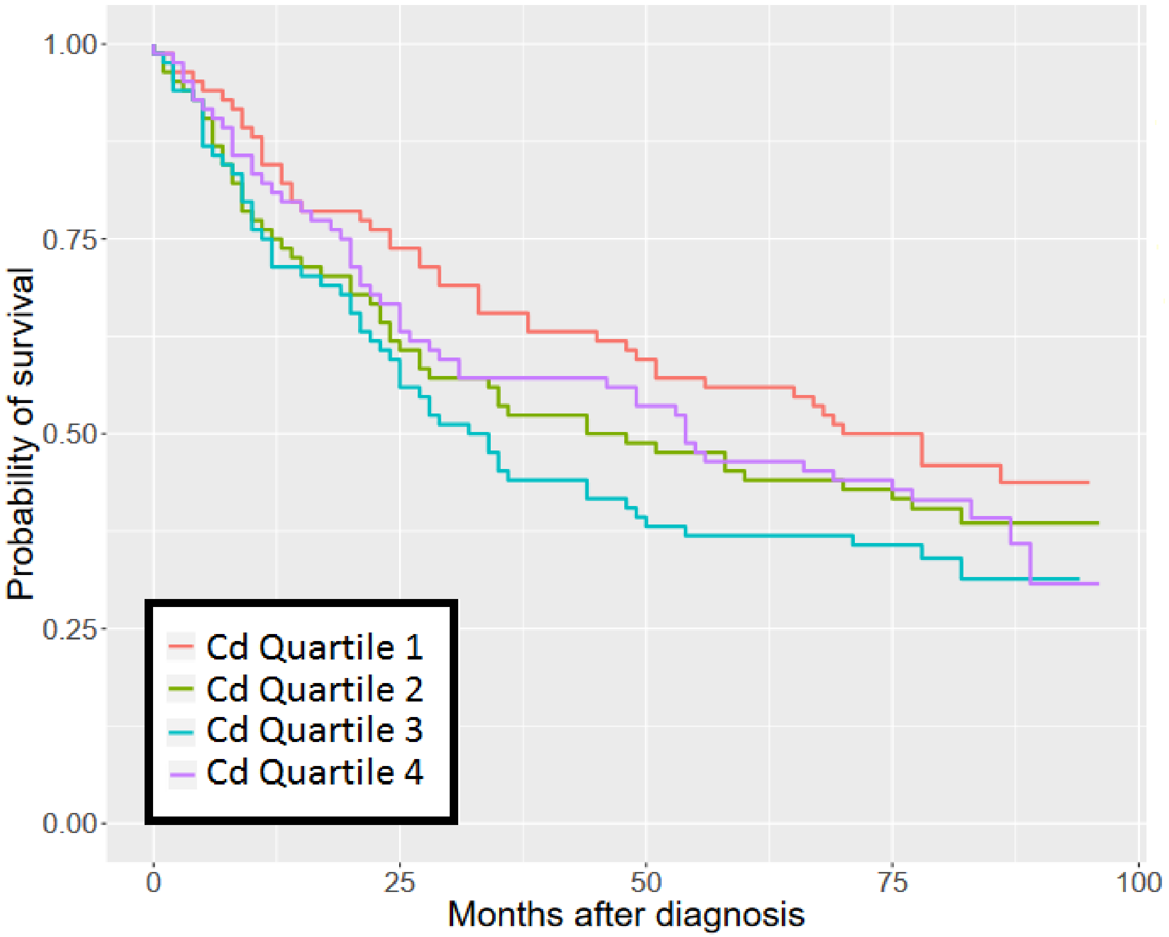

Figure S2. 96-months overall survival by cadmium levels in the group of all lung cancer.

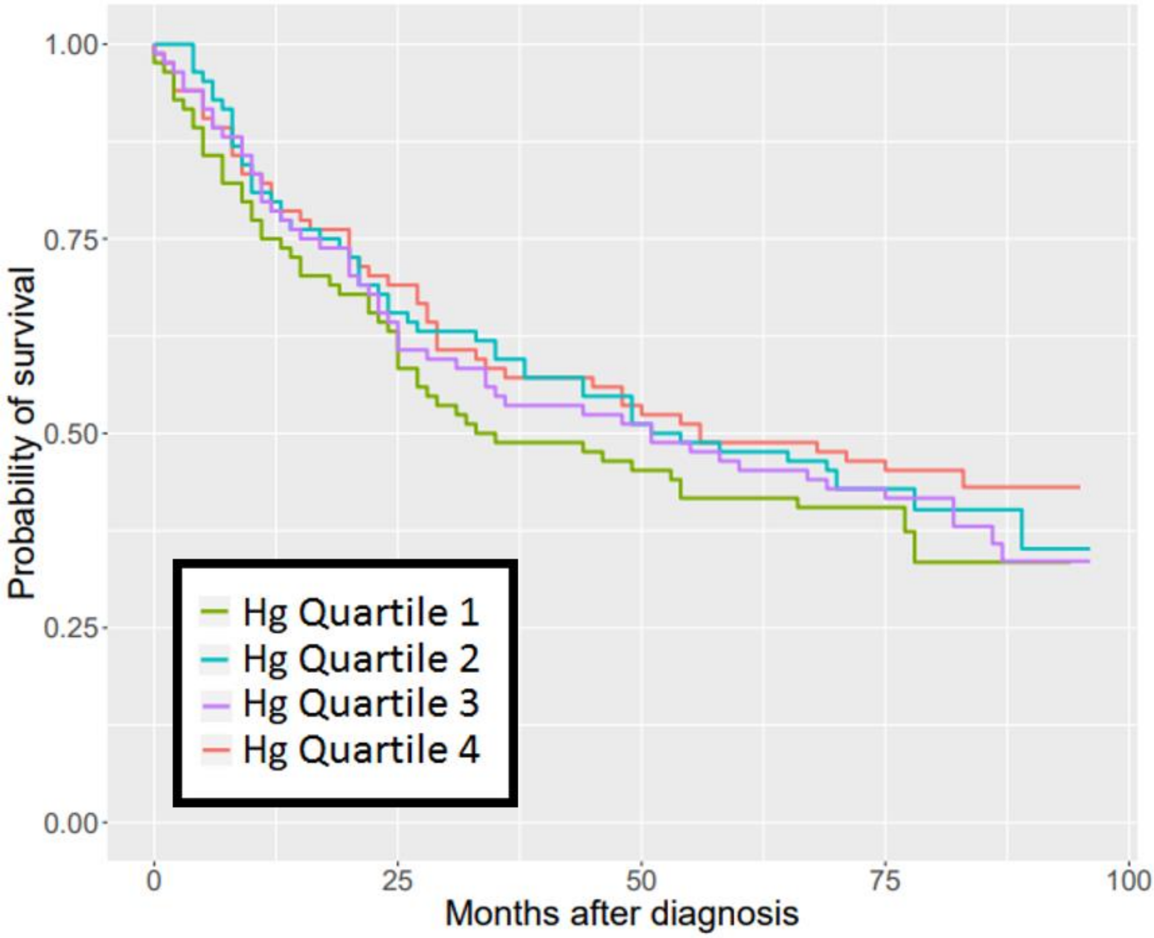

Figure S3. 96-months overall survival by mercury levels in the group of all lung cancer.

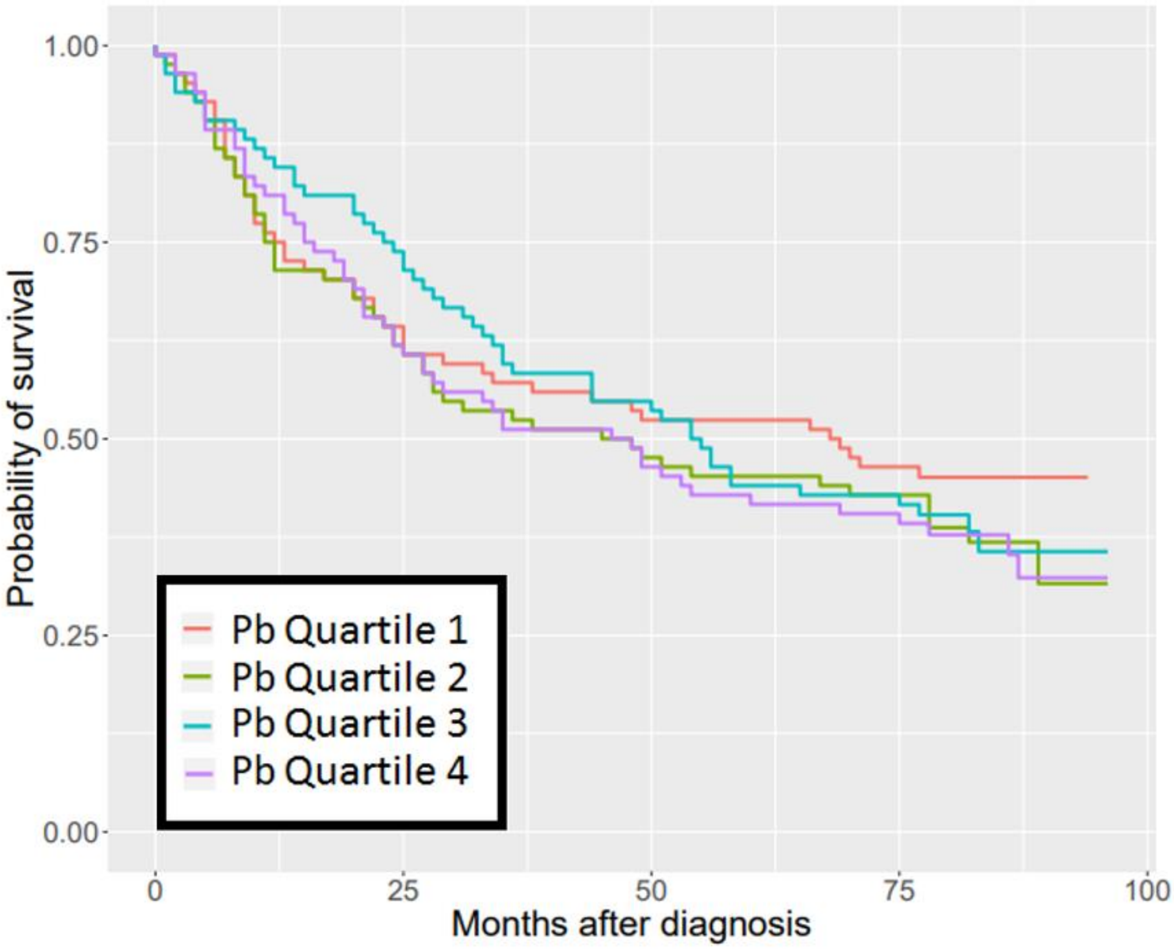

Figure S4. 96-months overall survival by lead levels in the group of all lung cancer.

Table S1. Mean levels of heavy metals by analysed categories.

Subgroup	N	Mean As level (range) [μg/L]	Mean Cd level (range) [μg/L]	Mean Hg level (range) [μg/L]	Mean Pb level (range) [μg/L]
**All**	336	1.02 (0.25–6.69)	1.45 (0.23–7.77)	1.02 (0.01–6.09)	25.29 (5.91–149.44)
**Male**	222	1.02 (0.28–6.69)	1.35 (0.23–6.99)	1.07 (0.02–6.09)	28.85 (5.91–149.44)
**Female**	114	1.01 (0.25–4.43)	1.63 (0.24–7.77)	0.93 (0.01–5.35)	18.34 (6.07–62.47)
**Age**					
**<=60**	124	0.92 (0.25–4.16)	1.79 (0.23–7.77)	0.90 (0.01–3.09)	28.75 (7.40–149.44)
**>60**	212	1.08 (0.28–6.69)	1.25 (0.24–6.04)	1.09 (0.02–6.09)	23.26 (5.91–94.83)
**Smoking**[1]					
**Yes**	315	1.01 (0.25–6.69)	1.51 (0.25–7.77)	1.01 (0.01–6.09)	25.78 (6.07–149.44)
**No**	21	1.13 (0.37–3.20)	0.57 (0.23–2.99)	1.20 (0.02–3.63)	17.83 (5.91–47.40)
**Heavy metal**					
**Quartile I**	84	0.50 (0.25–0.60)	0.47 (0.23–0.67)	0.27 (0.01–0.44)	11.85 (5.91–15.57)
**Quartile II**	84	0.69 (0.60–0.79)	0.90 (0.68–1.13)	0.59 (0.44–0.74)	17.96 (15.61–20.80)
**Quartile III**	84	0.93 (0.78–1.15)	1.43 (1.13–1.86)	0.99 (0.75–1.30)	25.07 (20.88–30.32)
**Quartile IV**	84	1.96 (1.16–6.69)	2.98 (1.87–7.77)	2.23 (1.31–6.09)	46.26 (30.45–149.44)
**Radiotherapy**					
**Yes**	102	1.00 (0.28–3.17)	1.46 (0.24–7.77)	1.07 (0.03–6.09)	26.52 (7.40–149.44)
**No**	234	1.03 (0.25–6.69)	1.44 (0.23–6.04)	1.00 (0.01–5.35)	24.75 (5.91–149.44)
**Chemotherapy**					
**Yes**	101	1.07 (0.25–6.69)	1.47 (0.25–6.04)	1.06 (0.12–6.09)	25.71 (6.96–85.92)
**No**	235	1.00 (0.28–4.16)	1.44 (0.23–7.77)	1.00 (0.01–5.35)	25.10 (5.91–149.44)
**Stage**					

**I**		153	0.99 (0.28–4.43)	1.37 (0.23–7.77)	0.94 (0.02–3.63)	23.93 (5.91–85.92)
	**IA**	89	0.93 (0.33–4.43)	1.50 (0.23–7.77)	0.96 (0.02–3.63)	23.63 (6.87–85.92)
	**IB**	64	1.07 (0.28–4.16)	1.18 (0.29–4.08)	0.90 (0.10–3.35)	24.34 (5.91–76.47)
**II**		91	1.05 (0.35–3.93)	1.52 (0.28–6.04)	1.13 (0.01–5.35)	25.74 (6.07–62.47)
	**IIA**	28	0.93 (0.35–3.93)	1.30 (0.28–4.35)	1.21 (0.05–4.67)	28.60 (6.07–58.28)
	**IIB**	63	1.10 (0.40–3.13)	1.62 (0.28–6.04)	1.10 (0.01–5.35)	24.46 (9.97–62.47)
**III**		76	1.11 (0.25–6.69)	1.46 (0.27–6.99)	1.09 (0.16–6.09)	28.31 (6.96–149.44)
	**IIIA**	55	1.08 (0.25–3.37)	1.46 (0.27–6.99)	1.03 (0.12–6.09)	31.82 (7.19–149.44)
	**IIIB**	20	1.21 (0.42–6.69)	1.44 (0.30–3.98)	1.24 (0.12–5.24)	19.30 (6.96–36.13)
	**IIIC**	1	0.74	1.76	1.31	14.92
**IV**		16	0.72 (0.44–1.29)	1.72 (0.25–5.68)	0.83 (0.19–2.56)	21.35 (10.28–44.41)
	**IVA**	15	0.71 (0.44–1.29)	1.69 (0.25–5.68)	0.82 (0.19–2.56)	21.68 (10.28–44.41)
	**IVB**	1	0.79	2.12	1.04	16.50

[1]Smoking includes both, current and past smokers.

Table S2. Hazard ratios and 95% confidence intervals for various factors on survival from lung cancer depending on to arsenic levels.

	**Univariate Cox Regression Models**			**Multivariate Cox Regression Models**		
**Risk factor**	**Hazard ratio**	**95% CI**	***p*-value**	**Hazard ratio**	**95% CI**	***p*-value**
**Age**						
**<=60**	1			1		
**>60**	1.14	0.86–1.52	0.36	1.26	0.94–1.70	0.12
**Sex**						
**Male**	1.33	0.98–1.79	0.07	1.21	0.89–1.64	0.23
**Female**	1			1		
**Stage**						
**I**	1			1		
**II**	1.60	1.13–2.26	<0.01	1.49	1.04–2.13	<0.01
**III**	2.78	1.97–3.92	<0.01[1]	2.10	1.40–3.16	<0.01[1]
**IV**	4.59	2.57–8.17	<0.01	3.79	2.05–7.02	<0.01[1]
**Radiotherapy**						
**Yes**	2.18	1.64–2.89	<0.01	1.51	1.09–2.11	0.01
**No**	1			1		
**Chemotherapy**						
**Yes**	1.52	1.14–2.02	<0.01	1.12	0.81–1.54	0.49
**No**	1			1		
**Smoker**						
**Yes**	1.53	0.81–2.90	0.19	1.23	0.65–2.35	0.52
**No**	1			1		
**Arsenic**						
**Quartile I [µg/L] (0.25–0.60)**	1			1		
**Quartile II [µg/L] (>0.60–0.79)**	1.09	0.74–1.63	0.66	1.01	0.68–1.52	0.95
**Quartile III [µg/L] (>0.79–1.15)**	1.23	0.84–1.80	0.29	1.11	0.76–1.64	0.59
**Quartile IV [µg/L] (>1.15–6.69)**	1.11	0.74–1.65	0.61	0.99	0.66–1.48	0.94

[1] Proportional Hazard Requirement is not achieved

Table S3. Hazard ratios and 95% confidence intervals for various factors on survival from lung cancer depending on to cadmium levels.

	**Univariate Cox Regression Models**			**Multivariate Cox Regression Models**		
**Risk factor**	**Hazard ratio**	**95% CI**	***p*-value**	**Hazard ratio**	**95% CI**	***p*-value**
**Age**						
**<=60**	1			1		
**>60**	1.14	0.86–1.52	0.36	1.27	0.94–1.7	0.12
**Sex**						
**Male**	1.33	0.98–1.79	0.07	1.25	0.92–1.70	0.16
**Female**	1			1		
**Stage**						
**I**	1			1		
**II**	1.60	1.13–2.26	<0.01	1.44	1.01–2.05	0.05
**III**	2.78	1.97–3.92	<0.01[1]	2.01	1.34–3.02	<0.01[1]
**IV**	4.59	2.57–8.17	<0.01	3.70	2.02–6.76	<0.01[1]
**Radiotherapy**						
**Yes**	2.18	1.64–2.89	<0.01	1.63	1.17–2.26	<0.01
**No**	1			1		
**Chemotherapy**						
**Yes**	1.52	1.14–2.02	<0.01	1.11	0.81–1.52	0.53
**No**	1			1		
**Smoker**						
**Yes**	1.53	0.81–2.90	0.19	0.97	0.49–1.92	0.92
**No**	1			1		
**Cadmium**						
**Quartile I [μg/L] (0.23–0.67)**	1			1		
**Quartile II [μg/L] (>0.67–1.13)**	1.27	0.85–1.88	0.25	1.39	0.91–2.11	0.13
**Quartile III [μg/L] (>1.13–1.86)**	**1.53**	1.04–2.27	**0.03**	**1.56**	1.02–2.36	**0.04**
**Quartile IV [μg/L] (>1.86–7.77)**	1.24	0.83–1.85	0.28	1.37	0.89–2.10	0.15

[1] Proportional Hazard Requirement is not achieved

Table S4. Hazard ratios and 95% confidence intervals for various factors on survival from lung cancer depending on to mercury levels.

	Univariate Cox Regression Models			Multivariate Cox Regression Models		
**Risk factor**	**Hazard ratio**	**95% CI**	***p*-value**	**Hazard ratio**	**95% CI**	***p*-value**
**Age**						
**<=60**	1			1		
**>60**	1.14	0.86–1.52	0.36	1.31	0.97–1.76	0.08
**Sex**						
**Male**	1.33	0.98–1.79	0.07	1.29	0.94–1.77	0.11
**Female**	1			1		
**Stage**						
**I**	1			1		
**II**	1.60	1.13–2.26	<0.01	1.51	1.06–2.16	0.02
**III**	2.78	1.97–3.92	<0.01[1]	2.08	1.38–3.13	<0.01[1]
**IV**	4.59	2.57–8.17	<0.01	3.73	2.03–6.86	<0.01[1]
**Radiotherapy**						
**Yes**	2.18	1.64–2.89	<0.01	1.59	1.15–2.22	<0.01
**No**	1			1		
**Chemotherapy**						
**Yes**	1.52	1.14–2.02	<0.01	1.19	0.86–1.64	0.30
**No**	1			1		
**Smoker**						
**Yes**	1.53	0.81–2.90	0.19	1.11	0.58–2.13	0.75
**No**	1			1		
**Mercury**						
**Quartile I [µg/L] (0.01–0.44)**	1.29	0.88–1.92	0.20	**1.55**	1.03–2.34	**0.04**
**Quartile II [µg/L] (>0.44–0.74)**	1.10	0.74–1.63	0.64	1.20	0.80–1.79	0.38
**Quartile III [µg/L] (>0.74–1.30)**	1.17	0.79–1.73	0.44	**1.49**	0.99–2.22	**0.05**
**Quartile IV [µg/L] (>1.30–6.09)**	1			1		

[1] Proportional Hazard Requirement is not achieved

Table S5. Hazard ratios and 95% confidence intervals for various factors on survival from lung cancer depending on to lead levels.

	Univariate Cox Regression Models			Multivariate Cox Regression Models		
**Risk factor**	**Hazard ratio**	**95% CI**	***p*-value**	**Hazard ratio**	**95% CI**	***p*-value**
**Age**						
**<=60**	1			1		
**>60**	1.14	0.86–1.52	0.36	1.26	0.93–1.70	0.13
**Sex**						
**Male**	1.33	0.98–1.79	0.07	1.12	0.80–1.58	0.50
**Female**	1			1		
**Stage**						
**I**	1			1		
**II**	1.60	1.13–2.26	<0.01	1.45	1.02–2.07	0.04
**III**	2.78	1.97–3.92	<0.01[1]	2.12	1.41–3.19	<0.01[1]
**IV**	4.59	2.57–8.17	<0.01	3.99	2.17–7.35	<0.01[1]
**Radiotherapy**						
**Yes**	2.18	1.64–2.89	<0.01	1.58	1.13–2.19	<0.01
**No**	1			1		
**Chemotherapy**						
**Yes**	1.52	1.14–2.02	<0.01	1.11	0.81–1.53	0.52
**No**	1			1		
**Smoker**						
**Yes**	1.53	0.81–2.90	0.19	1.22	0.64–2.34	0.55
**No**	1			1		
**Lead**						
**Quartile I [μg/L] (5.91–15.57)**	1			1		
**Quartile II [μg/L] (>15.57–20.80)**	1.21	0.82–1.80	0.34	1.37	0.90–2.10	0.14
**Quartile III [μg/L] (>20.80–30.32)**	1.10	0.74–1.63	0.65[1]	1.25	0.81–1.93	0.32
**Quartile IV [μg/L] (>30.32–149.44)**	1.23	0.83–1.82	0.31	1.18	0.76–1.82	0.47

[1] Proportional Hazard Requirement is not achieved

Table S6. Hazard ratios and 95% confidence intervals for various factors on survival from lung cancer depending on to cadmium levels for patients with lung cancer with stage IB–IVB.

	Univariable Cox Regression Models			Multivariable Cox Regression Models		
**Risk factor**	**Hazard ratio**	**95% CI**	***p*-value**	**Hazard ratio**	**95% CI**	***p*-value**
**Age**						
**<=60**	1			1		
**>60**	1.23	0.89–1.69	0.21	1.23	0.88–1.72	0.22
**Sex**						
**Male**	1.07	0.77–1.51	0.68	1.07	0.76–1.51	0.69
**Female**	1			1		
**Radiotherapy**						
**Yes**	1.70	1.25–2.32	<0.01	1.73	1.26–2.36	<0.01
**No**	1			1		
**Chemotherapy**						
**Yes**	1.14	0.84–1.56	0.40	1.08	0.78–1.50	0.65
**No**	1			1		
**Smoker**						
**Yes**	0.93	0.41–2.09	0.85	0.82	0.35–1.90	0.64
**No**	1			1		
**Cadmium**						
**Quartile I [µg/L] (0.25–0.70)**	1			1		
**Quartile II [µg/L] (>0.70–1.13)**	1.17	0.75–1.81	0.48	1.25	0.80–1.96	0.33
**Quartile III [µg/L] (>1.13–1.69)**	1.38	0.90–2.12	0.14	1.52	0.97–2.37	0.07
**Quartile IV [µg/L] (>1.69–6.99)**	1.07	0.69–1.65	0.77	1.21	0.76–1.93	0.41

Table S7. Hazard ratios and 95% confidence intervals for various factors on survival from lung cancer depending on to cadmium levels for patients with lung cancer with stage IA.

	**Univariable Cox Regression Models**			**Multivariable Cox Regression Models**		
**Risk factor**	**Hazard ratio**	**95% CI**	***p*-value**	**Hazard ratio**	**95% CI**	***p*-value**
**Age**						
**<=60**	1			1		
**>60**	0.98	0.51–1.89	0.96	1.48	0.72–3.06	0.29
**Sex**						
**Male**	1.75	0.90–3.41	0.10	1.86	0.90–3.84	0.09
**Female**	1			1		
**Radiotherapy**						
**Yes**	3.89	1.77–8.58	<0.01	7.45	2.73–20.38	<0.01
**No**	1			1		
**Chemotherapy**						
**Yes**	3.35	1.58–7.11	<0.01	2.46	1.03–5.83	0.04
**No**	1			1		
**Smoker**						
**Yes**	1.62	0.58–4.56	0.36	0.50	0.13–1.82	0.29
**No**	1			1		
**Cadmium**						
**Quartile I [µg/L] (0.23–0.57)**	1			1		
**Quartile II [µg/L] (>0.57–1.11)**	1.24	0.48–3.21	0.66	3.55	1.02–12.35	0.05[1]
**Quartile III [µg/L] (>1.11–1.97)**	0.96	0.35–2.66	0.94	2.41	0.64–9.06	0.19[1]
**Quartile IV [µg/L] (>1.97–7.77)**	**2.74**	1.15–6.50	**0.02**	**7.36**	2.14–25.25	**<0.01**

[1] Proportional Hazard Requirement is not achieved
